# Reply to Dr. Reynolds in Relation to an Eruptive Disease at Hornelsville

**Published:** 1854-09

**Authors:** Frank H. Hamilton


ART. V.—*Reply to Dr. Reynolds in relation to an eruptive disease at Hornelsville.* By FRANK H. HAMILTON.

My excelleht friend Dr. Reynolds of Dansville differs with me in relation to two cases of eruptive disease which I was called to see at Hornelsville, Steuben Co., N. Y. on the 20th of April last, and he has sought to defend his opinion by a very ingenious argument published in a daily paper. I do not suppose your readers care very much whether I was right or wrong in my diagnosis, and it is not for their benefit so much as for my own that I trouble you. Dr. Reynolds is a gentlemen whose opinions are entitled to, and will command, respect, and I cannot properly in justice to myself or in courtesy to him, avoid a reply.

During several weeks or months an eruptive disease had been prevailing at Hornelsville, in relation to the character of which, many very respectable physicians were divided, a part calling it variola or varioloid and a part varicella. The citizens, were also, divided in opinion, and what with their pecuniary interest and their bodily interest, both of which were involved in the question of its name, they had become, I am told, very much excited.

The disease was still progressing when I received a letter from Hornelsville requesting my attendance at the house of Mr. Wilson, a very respectable citizen of that place. On the next or following day I went to Hornelsville, and in company with Dr. Wisewell, the attending physician, called at Mr. Wilson's house.

Mr. Wilson requested me to examine two children belonging to his family and give my opinion as to the character of the disease from which they were now recovering.

The following is an account of the cases, copied from notes taken in the house and while the examination was progressing.

*Case first*—John, son of Stephen L. Wilson, aged two years. Healthy child, never had any eruptive disease until now. Has never been vaccinated. I found no vaccine scar, and the father said, he knew the fact beyond question that he had never been vaccinated.

On Wednesday, April 12th an eruption commenced without any previous illness, on the front of his neck. Dr. Wisewell was called and ordered him castor oil. During the day he was a little peevish and eat rather less than usual. In the evening the eruption had extended to his back and forehead.

Thursday, the eruption had reached his face. The child was playful and seemed well. The eruption continued generally to spread, and on

Sunday, (4th day,) the points which appeared first had matured and completely desiccated.

Thursday, (8th day) the day of my visit, no eruption or traces of an eruption was visible upon the front of the neck, upon the face or forehead. About fifteen or twenty reddish points upon the belly, of the size of pin heads. Upon the back were about an equal number of spots, varying in size, from half a line to two lines in diameter, of a dark brown color and dry. Upon the bottom of the feet, were four recent eruptions, of about half a line in diameter and filled with serum. No indentation or scar was left any where upon the body, The child was perfectly well and had been so from the first day of the eruption.

*Case Second*—Elizabeth Mathews, daughter of Bridget Mathews, aged 3 years and 9 months. She was a healthy child and not subject to eruptions. She was vaccinated two years ago. Has a distinct vaccine scar on her arm.

Without any previous illness the eruption appeared on Wednesday morning. It pursued nearly the same course as with the other child, showing itself first on the neck, face and head, then on the body, &c. The mother says the eruptions on the neck began to dry on the third day.

Thursday, the eighth day, and the day of my visit, there were remaining a few small red and brown points on various parts of the body, of the size of pin heads. Those upon the body were larger and five or six of them were quite four lines in diameter, irregular in form, covered with a thin, scaly crust, and surrounded witn a slight, unequal areola. No where on the body could a depression or scar be found.

The child had not been ill one hour during the eruption, nor had she taken medicine. She had played out and in doors, and slept and eat as usual.

These were the only cases I was asked to examine, and they were the only cases I saw. I called them varicella. My friend, Dr. Reynolds, calls them variola or varioloid.

I have read the argument of Dr. Reynolds carefully, but it has failed to convince me that I am in error. Whether variola actually existed in Hornelsville, or not I do not know. Its absence or its presence in the town would not have changed my opinion of these cases; for varicella might exist, and even prevail epidemically, under either circumstance.

For any inferences which other gentlemen may have drawn, I am not responsible. Dr. Wisewell, regarded my opinion on these cases as decisive of the whole question, whether variola was present in Hornelsville or not. Dr. Wisewell was familiar with the other cases and could judge. He is a shrewd, intelligent man, and will, I presume take care of himself.

